# Supplementary material for: Practical diagnosis of cirrhosis in non-alcoholic fatty liver disease using currently available non-invasive fibrosis tests
Source: Nat Commun. 2023 Aug 26;14:5219. doi: 10.1038/s41467-023-40328-4 (PMC10460420; doi:10.1038/s41467-023-40328-4)

Practical diagnosis of cirrhosis in non-alcoholic fatty liver disease using currently available non-invasive fibrosis tests

J Boursier, M Roux, C Costentin, J Chaigneau, C Fournier-Poizat, A Trylesinski, CM Canivet, S Michalak, B Le Bail, V Paradis, P Bedossa, N Sturm, V de Ledinghen, PN Newsome

SUPPLEMENTARY INFORMATION

Supplementary Table s1: Non-invasive tests of liver fibrosis. 2

Supplementary Table s2: Accuracy of fibrosis tests for the diagnosis of cirrhosis using F34 and F4 thresholds in the derivation set. 3

Supplementary Table s3: Accuracy of fibrosis tests for the diagnosis of cirrhosis using F34 and F4 thresholds in the validation set. 4

Supplementary Table s4: Contingency table showing patients as a function of fibrosis stage on liver biopsy and the four diagnostic groups defined by the study algorithm. 5

Supplementary Table s5: Diagnostic accuracy of the EASL pathway, the Agile3+/4 classification and the study algorithm. 6

Supplementary Table s6: Diagnostic accuracy of the study algorithm and the Agile3+/4 classification for the binary diagnosis of cirrhosis in the validation set. 7

Supplementary Table s7: TRIPOD Checklist for prediction model development and validation. 8

Supplementary Figure s1: Flow chart of the study 9

Supplementary Figure s2: Rate of cirrhosis as a function of fibrosis tests results. 10

Supplementary Figure s3: Rates of false negatives, true negatives, true positives, and false positives for the diagnosis of cirrhosis using F4 thresholds (panel s3a) or F34 thresholds (panel s3b) in the derivation set. 12

Supplementary Figure s4: Rates of false negatives, true negatives, true positives, and false positives for the diagnosis of cirrhosis using F4 thresholds (panel s4a) or F34 thresholds (panel s4b) in the validation set. 13

Supplementary Figure s5: Correlation between FibroMeter^{V3G} and CirrhoMeter^{V3G}, and between Agile 3+ and Agile4 in the derivation and validation sets. 14

Supplementary Figure s6: Fibrosis stages as a function of 16 subgroups defined by the crossing of Agile3+/4 and FibroMeter^{V3G}/CirrhoMeter^{V3G} (FM/CM) classifications in the derivation set. 15

Supplementary Figure s7: Diagnostic pathway proposed by the latest EASL guidelines for the specialist area, based on agreement between non-invasive tests. 16

Supplementary Figure s8: Area of fibrosis measured by morphometry as a function of fibrosis stage. 17

Supplementary Figure s9: Area of fibrosis measured by morphometry on liver biopsies from Angers centre (validation set). 18

Supplementary Figure s10: Calibration of the predicted risk of cirrhosis by Agile4 and CirrhoMeter^{V3G} in the validation set. 20

Supplementary Table s1: Non-invasive tests of liver fibrosis.

Fibrosis test	Targeted for [a]	Formula	F34 Thresholds [b]		F4 Thresholds [c]	
			Rule-out	Rule-in	Rule-out	Rule-in
FIB4	Advanced fibrosis F34	(Age [years] x AST [IU/l]) / (platelets [G/l] x ALT $(IU/l)^{1/2}$)	<1.30 [(1)]	>2.67 [(1)]	<1.24 [d]	>3.22 [d]
NAFLD fibrosis score	Advanced fibrosis F34	-1.675 + (0.037 x age [years]) + (0.094 x BMI [kg/m^2]) + (1.13 x impaired fasting glucose / diabetes [yes: 1, no: 0]) + (0.99 x AST/ALT ratio) – (0.013 x platelet [G/l]) – (0.66 x albumin [g/dl]).	<-1.455 [(2)]	>0.676 [(2)]	<-1.107 [d]	>1.302 [d]
FibroMeter^{V3G}	Advanced fibrosis F34	Patented formula calculated by Echosens (age, sex, urea, AST, gammaGT, prothrombin time, platelets, alpha2-macroglobulin)	<0.31 [d]	>0.76 [d]	<0.44 [d]	>0.91 [d]
CirrhoMeter^{V3G}	Cirrhosis F4	Patented formula calculated by Echosens (age, sex, urea, AST, gammaGT, prothrombin time, platelets, alpha2-macroglobulin)	<0.007 [d]	>0.11 [d]	<0.017 [d]	>0.40 [d]
VCTE	-	-	8.0 kPa [(3, 4)]	12.0 kPa [(3, 4)]	<8.8 kPa [d]	>21.0 kPa [d]
Agile3+	Advanced fibrosis F34	$e^R / (1 + e^R)$ with R = 7.50139 – (15.42498 / VCTE $[kPa]^{1/2}$) – (0.01378 × platelets [G/l]) − (1.41149 × AST/ALT $ratio^{-1}$) – (0.53281 × sex [female: 0, male: 1]) + (0.41741 × diabetes status [no: 0, yes: 1])	<0.451 [(5)]	≥0.679 [(5)]	<0.581 [d]	>0.930 [d]
Agile4	Cirrhosis F4	$e^R / (1 + e^R)$ with R = − 3.92368 + (2.29714 x ln(VCTE [kPa])) – (0.00902 × platelets [G/l]) − (0.98633 × AST/ALT $ratio^{-1}$) – (0.38581 × sex [female: 0, male: 1]) + (1.08636 × diabetes status [no: 0, yes: 1]) + (0.03018 x age [years])	<0.024 [d]	>0.148 [d]	<0.087 [d]	>0.474 [d]

ALT: alanine aminotransferase; AST: aspartate aminotransferase; BMI: body mass index; gammaGT: gamma glutamyl transferase; LSM: liver stiffness measurement; VCTE: vibration-controlled transient elastography

[a] Diagnostic target for which the fibrosis test has been developed

[b] Thresholds dedicated to the diagnosis of advanced liver fibrosis in NAFLD

[c] Thresholds dedicated to the diagnosis of cirrhosis in NAFLD

[d] Calculated in the derivation set of the study (90% sensitivity and 90% specificity for advanced fibrosis; 90% sensitivity and 95% specificity for cirrhosis), with further validation in the validation set

(1) Shah *et al*, PMID 19523535; (2) Angulo *et al*, PMID 17393509 ; (3) Papatheodoridi *et al*, PMID 33307138 ; (4) Mozes *et al*, PMID 34001645 ; (5) Pennisi *et al*, PMID 35842119

Supplementary Table s2: Accuracy of fibrosis tests for the diagnosis of cirrhosis using F34 and F4 thresholds in the derivation set.

Threshold	Fibrosis test	Rule out cirrhosis				Grey zone (RoP, %)	Rule in cirrhosis			
		Threshold	RoP (%)	Se (%)	NPV (%)		Threshold	RoP (%)	Spe (%)	PPV (%)
Dedicated to F34	FIB4	<1.30	35.1	92.6	97.1	47.9	>2.67	17.0	87.9	38.5
	NFS	<-1.455	48.4	87.6	96.4	36.8	>0.676	14.8	90.3	43.4
	FMV3G	<0.31	29.4	97.5	98.8	48.1	>0.76	22.6	83.4	36.5
	VCTE	<8.0	45.8	95.0	98.5	26.9	>12.0	27.3	79.8	36.1
	Agile3+	<0.451	52.1	94.2	98.5	16.1	≥0.679	31.9	75.8	34.5
	CMV3G	<0.007	21.2	94.2	96.2	55.2	>0.11	23.6	82.3	35.4
	Agile4	<0.024	36.9	97.5	99.1	34.4	>0.148	28.7	79.2	37.6
Dedicated to F4	FIB4	<1.24	43.9	90.1	96.9	47.5	>3.22	8.6	94.5	45.3
	NFS	<-1.107	44.7	90.1	96.9	45.3	>1.302	10.0	94.7	54.0
	FMV3G	<0.44	45.4	90.9	97.2	45.5	>0.91	9.1	95.2	54.4
	VCTE	<8.8	51.8	90.1	97.3	36.9	>21.0	11.2	94.5	58.2
	Agile3+	<0.581	61.4	90.1	97.8	27.2	>0.930	11.5	94.5	59.0
	CMV3G	<0.017	41.2	90.1	96.7	48.7	>0.40	10.1	94.7	54.5
	Agile4	<0.087	61.8	90.1	97.8	26.5	>0.474	11.7	94.5	59.8

RoP: rate of patients included in the interval; Se: sensitivity; Spe: specificity; NPV: negative predictive value; PPV positive predictive value

Supplementary Table s3: Accuracy of fibrosis tests for the diagnosis of cirrhosis using F34 and F4 thresholds in the validation set.

Threshold	Fibrosis test	Rule out cirrhosis				Grey zone (RoP, %)	Rule in cirrhosis			
		Threshold	RoP (%)	Se (%)	NPV (%)		Threshold	RoP (%)	Spe (%)	PPV (%)
Dedicated to F34	FIB4	<1.30	34.3	95.6	98.7	48.7	>2.67	17.0	86.0	25.4
	NFS	<-1.455	46.0	82.4	96.3	42.4	>0.676	11.6	92.4	40.7
	FMV3G	<0.31	29.7	97.1	99.0	51.6	>0.76	18.7	85.5	30.0
	VCTE	<8.0	42.2	98.5	99.7	26.9	>12.0	30.9	75.3	27.9
	Agile3+	<0.451	52.0	97.1	99.4	18.7	≥0.679	29.3	77.5	30.9
	CMV3G	<0.007	23.1	97.1	98.8	55.5	>0.11	21.4	83.3	29.5
	Agile4	<0.024	37.8	98.5	99.6	35.1	>0.148	27.2	79.9	33.3
Dedicated to F4	FIB4	<1.24	41.7	91.2	97.9	49.4	>3.22	8.9	93.3	32.3
	NFS	<-1.107	42.8	85.3	96.6	51.0	>1.302	6.2	96.7	51.2
	FMV3G	<0.44	45.4	92.6	98.4	50.4	>0.91	4.2	97.6	48.3
	VCTE	<8.8	48.7	98.5	99.7	38.9	>21.0	12.4	92.4	44.2
	Agile3+	<0.581	60.9	95.6	99.3	26.7	>0.930	12.4	93.5	52.3
	CMV3G	<0.017	42.8	91.2	98.0	51.6	>0.40	5.6	97.1	53.8
	Agile4	<0.087	62.8	97.1	99.5	26.7	>0.474	10.5	95.2	58.9

RoP: rate of patients included in the interval; Se: sensitivity; Spe: specificity; NPV: negative predictive value; PPV positive predictive value

Supplementary Table s4: Contingency table showing patients as a function of fibrosis stage on liver biopsy and the four diagnostic groups defined by the study algorithm.

Green cells correspond to patients well classified by the algorithm and grey cells to those requiring liver biopsy. Yellow cells correspond to misclassified patients with only one fibrosis stage discrepancy, and orange cells to those with ≥2 stages discrepancy. Among misclassified patients (yellow and orange cells), 103/136 (76%) in the derivation set and 86/98 (88%) in the validation set were by only one fibrosis stage.

	**Fibrosis stage on liver biopsy**	**Diagnosis of the study algorithm**			
		**F0-2 (n)**	**Biopsy (n)**	**F34 (n)**	**F4 (n)**
**Derivation set**	F0	96	6	6	0
	F1	163	25	12	4
	F2	133	39	35	4
	F3	55	60	100	13
	F4	7	16	49	49
**Validation set**	F0	71	9	1	0
	F1	119	24	7	0
	F2	126	55	31	2
	F3	44	48	80	11
	F4	2	5	29	32

Supplementary Table s5: Diagnostic accuracy of the EASL pathway, the Agile3+/4 classification and the study algorithm.

The EASL pathway provides a three-class diagnosis (F0-2, grey zone and F34; see Supplementary Figure s7)

The Agile3+/4 classification provides a four-class diagnosis (F0-2, grey zone, F34 and F4; see Figure 1)

The study algorithm provides a four-class diagnosis (F0-2, grey zone, F34 and F4; see Figure 3)

**Study set**	**Algorithm**	**Algorithm diagnosis**								**All**		
		**F0-2**		**Grey zone**		**F34**		**F4**		**DA**	**Comparison (p value [a])**	
		Patients (%)	**DA (%)**	***Patients (%)***	**DA [b] (%)**	***Patients (%)***	**DA (%)**	***Patients (%)***	**DA (%)**	**(%)**	**vs Agile3+/4 classification**	**vs Study algorithm**
Derivation	EASL pathway	*45.8*	85.7	*17.7*	100.0	*36.6*	74.3	*0.0*	-	84.1	0.151	0.863
	Agile3+/4 classification	*52.1*	86.3	*16.1*	100.0	*20.2*	68.8	*11.7*	59.8	81.9	-	$3.0e^{-6}$
	Study algorithm	*52.1*	86.3	*16.7*	100.0	*23.2*	73.8	*8.0*	70.0	84.4	-	-
Validation	EASL pathway	*42.2*	91.2	*20.8*	100.0	*36.9*	72.4	*0.0*	-	86.1	0.037	1.000
	Agile3+/4 classification	*52.0*	87.3	*18.7*	100.0	*18.8*	65.6	*10.5*	58.9	82.6	-	$2.0e^{-6}$
	Study algorithm	*52.0*	87.3	*20.3*	100.0	*21.3*	73.6	*6.5*	71.1	85.9	-	-
All	EASL pathway	*44.2*	88.0	*19.1*	100.0	*36.7*	73.4	*0.0*	-	84.9	0.012	0.948
	Agile3+/4 classification	*52.0*	86.8	*17.2*	100.0	*19.6*	67.4	*11.2*	59.4	82.2	-	$4.4e^{-12}$
	Study algorithm	*52.0*	86.8	*18.3*	100.0	*22.3*	73.7	*7.3*	70.4	85.1	-	-

DA: diagnostic accuracy (rate of patients correctly classified)

[a] Two-sided McNemar test, no adjustment for multiple comparison

[b] By definition, all these patients undergo liver biopsy and the diagnostic accuracy is thus 100%

Supplementary Table s6: Diagnostic accuracy of the study algorithm and the Agile3+/4 classification for the binary diagnosis of cirrhosis in the validation set.

The study algorithm provides a four-class diagnosis (F0-2, grey zone, F34 and F4; see Figure 3). The Agile3+/4 classification provides also a four-class diagnosis (F0-2, grey zone, F34 and F4; see Figure 1). For this analysis, we considered the categories F0-2 and grey zone of the study algorithm and the Agile3+/4 classification as the rule-out zone for cirrhosis, the F3-4 category as the undetermined zone for cirrhosis (no discrimination between F4 and F3 patients), and the F4 category as the rule-in zone.

		**Study algorithm**	**Agile3+/4 classification**
Rule-out zone	Patients (%)	72.3	70.7
	Sensitivity (%)	89.7	88.7
	NPV (%)	98.6	98.4
Undetermined zone	Patients (%)	21.3	18.8
Rule-in zone	Patients (%)	6.5	10.5
	Specificity (%)	97.9	95.2
	PPV (%)	71.1	58.9

Supplementary Table s7: TRIPOD Checklist for prediction model development and validation.

Section/Topic	Item		Checklist Item	Page
**Title and abstract**				
Title	1	D;V	Identify the study as developing and/or validating a multivariable prediction model, the target population, and the outcome to be predicted.	1
Abstract	2	D;V	Provide a summary of objectives, study design, setting, participants, sample size, predictors, outcome, statistical analysis, results, and conclusions.	4
**Introduction**				
Background and objectives	3a	D;V	Explain the medical context (including whether diagnostic or prognostic) and rationale for developing or validating the multivariable prediction model, including references to existing models.	5
	3b	D;V	Specify the objectives, including whether the study describes the development or validation of the model or both.	5
**Methods**				
Source of data	4a	D;V	Describe the study design or source of data (e.g., randomized trial, cohort, or registry data), separately for the development and validation data sets, if applicable.	22
	4b	D;V	Specify the key study dates, including start of accrual; end of accrual; and, if applicable, end of follow-up.	22
Participants	5a	D;V	Specify key elements of the study setting (e.g., primary care, secondary care, general population) including number and location of centres.	22
	5b	D;V	Describe eligibility criteria for participants.	22
	5c	D;V	Give details of treatments received, if relevant.	NA
Outcome	6a	D;V	Clearly define the outcome that is predicted by the prediction model, including how and when assessed.	22-23
	6b	D;V	Report any actions to blind assessment of the outcome to be predicted.	22-23
Predictors	7a	D;V	Clearly define all predictors used in developing or validating the multivariable prediction model, including how and when they were measured.	23
	7b	D;V	Report any actions to blind assessment of predictors for the outcome and other predictors.	23
Sample size	8	D;V	Explain how the study size was arrived at.	22
Missing data	9	D;V	Describe how missing data were handled (e.g., complete-case analysis, single imputation, multiple imputation) with details of any imputation method.	22
Statistical analysis methods	10a	D	Describe how predictors were handled in the analyses.	24
	10b	D	Specify type of model, all model-building procedures (including any predictor selection), and method for internal validation.	24
	10c	V	For validation, describe how the predictions were calculated.	24
	10d	D;V	Specify all measures used to assess model performance and, if relevant, to compare multiple models.	24
	10e	V	Describe any model updating (e.g., recalibration) arising from the validation, if done.	NA
Risk groups	11	D;V	Provide details on how risk groups were created, if done.	NA
Development vs. validation	12	V	For validation, identify any differences from the development data in setting, eligibility criteria, outcome, and predictors.	22
**Results**				
Participants	13a	D;V	Describe the flow of participants through the study, including the number of participants with and without the outcome and, if applicable, a summary of the follow-up time. A diagram may be helpful.	Figure s1
	13b	D;V	Describe the characteristics of the participants (basic demographics, clinical features, available predictors), including the number of participants with missing data for predictors and outcome.	15
	13c	V	For validation, show a comparison with the development data of the distribution of important variables (demographics, predictors and outcome).	15
Model development	14a	D	Specify the number of participants and outcome events in each analysis.	15
	14b	D	If done, report the unadjusted association between each candidate predictor and outcome.	NA
Model specification	15a	D	Present the full prediction model to allow predictions for individuals (i.e., all regression coefficients, and model intercept or baseline survival at a given time point).	6-7
	15b	D	Explain how to the use the prediction model.	19
Model performance	16	D;V	Report performance measures (with CIs) for the prediction model.	7-8
Model-updating	17	V	If done, report the results from any model updating (i.e., model specification, model performance).	NA
**Discussion**				
Limitations	18	D;V	Discuss any limitations of the study (such as nonrepresentative sample, few events per predictor, missing data).	12
Interpretation	19a	V	For validation, discuss the results with reference to performance in the development data, and any other validation data.	12
	19b	D;V	Give an overall interpretation of the results, considering objectives, limitations, results from similar studies, and other relevant evidence.	10-12
Implications	20	D;V	Discuss the potential clinical use of the model and implications for future research.	11-12
**Other information**				
Supplementary information	21	D;V	Provide information about the availability of supplementary resources, such as study protocol, Web calculator, and data sets.	26
Funding	22	D;V	Give the source of funding and the role of the funders for the present study.	2

*Items relevant only to the development of a prediction model are denoted by D, items relating solely to a validation of a prediction model are denoted by V, and items relating to both are denoted D;V.

Supplementary Figure s1: Flow chart of the study

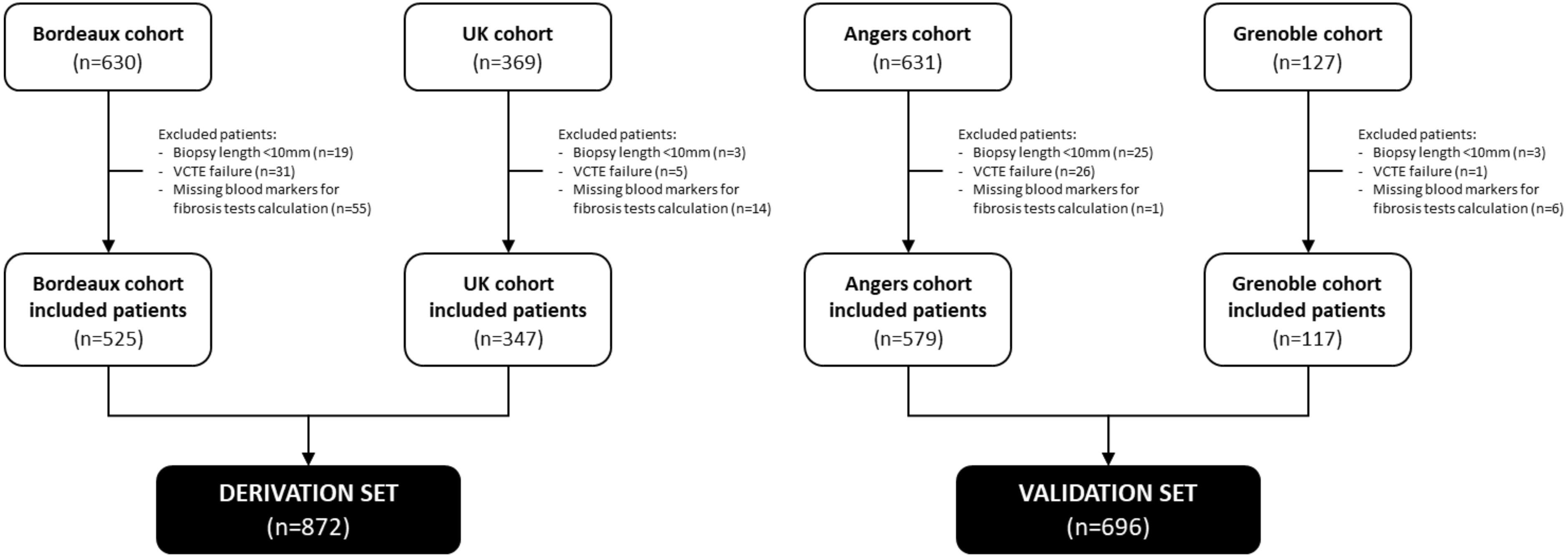

Phase 3 study design according to TRIPOD guidelines with development and validation sets using separate data

Supplementary Figure s2: Rate of cirrhosis as a function of fibrosis tests results.

The whole study population (n=1,568) was divided into 20 subgroups with the same number of patients and increasing fibrosis test result. Prevalence of fibrosis stages F0-2, F3 and F4 was calculated in each subgroup.

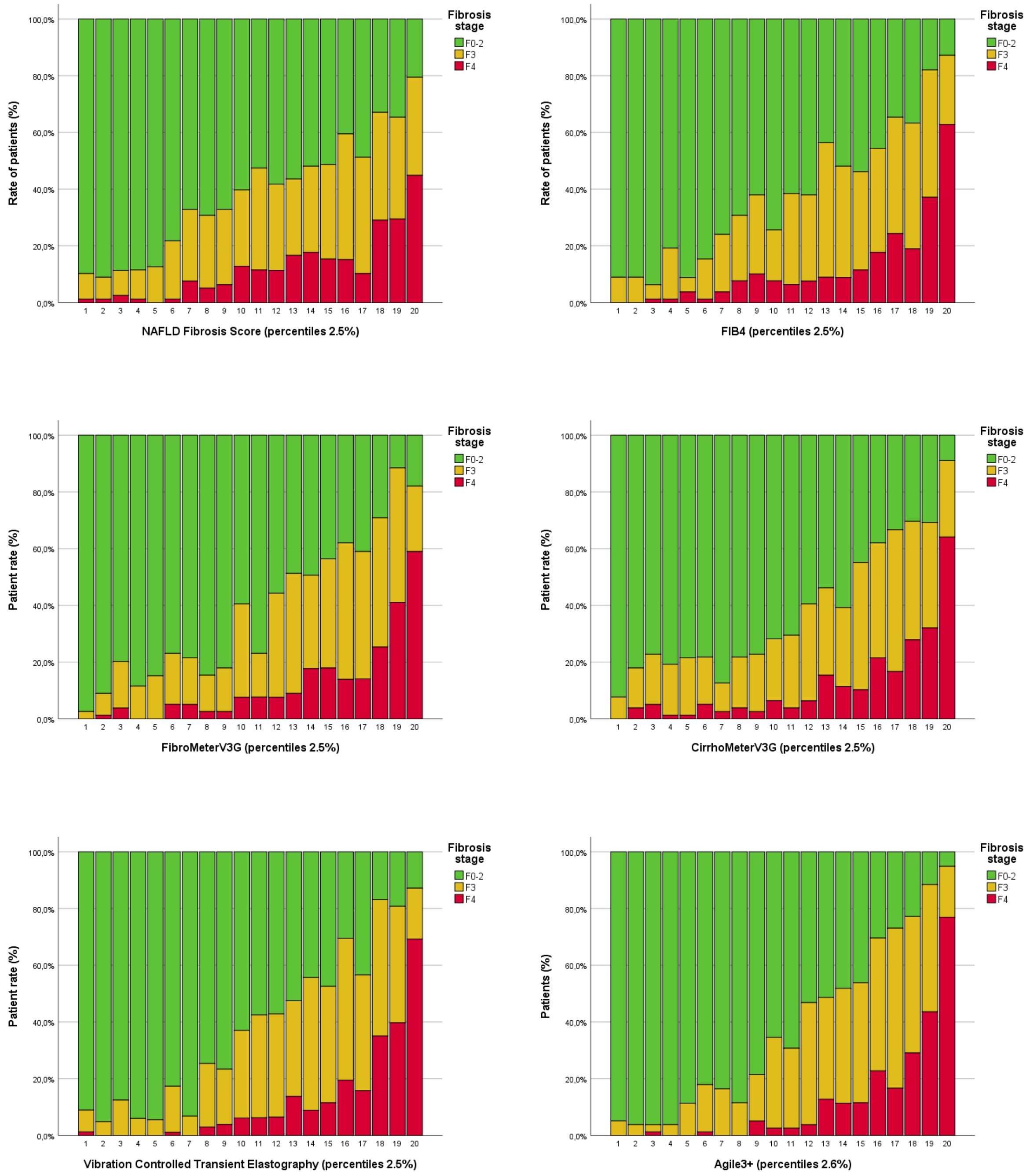

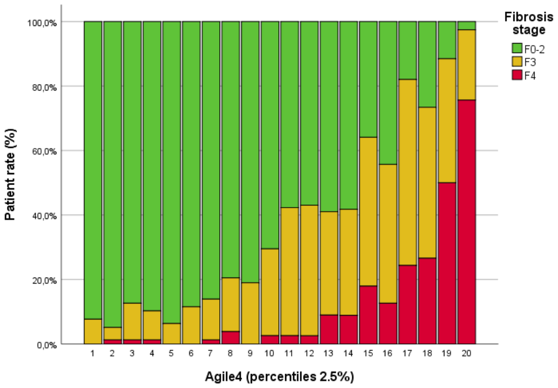

Fibrosis stage
F0-2
F3
F4
Patient rate (%)
100,0%
80,0%
60,0%
40,0%
20,0%
0,0%
1
2
3
4
5
6
7
8
9
10
11
12
13
14
15
16
17
18
19
20
Agile4 (percentiles 2.5%)

Supplementary Figure s3: Rates of false negatives, true negatives, true positives, and false positives for the diagnosis of cirrhosis using F4 thresholds (panel s3a) or F34 thresholds (panel s3b) in the derivation set.

Bars in dark and light green correspond to the patients ruled out for cirrhosis. Bars in light and dark grey correspond to the patients in the grey zone between the rule out and the rule in thresholds. Bars in light and dark orange correspond to the patients rule in for cirrhosis.

NFS: NAFLD fibrosis score; VCTE: vibration controlled transient elastography.

A: F4 Thresholds

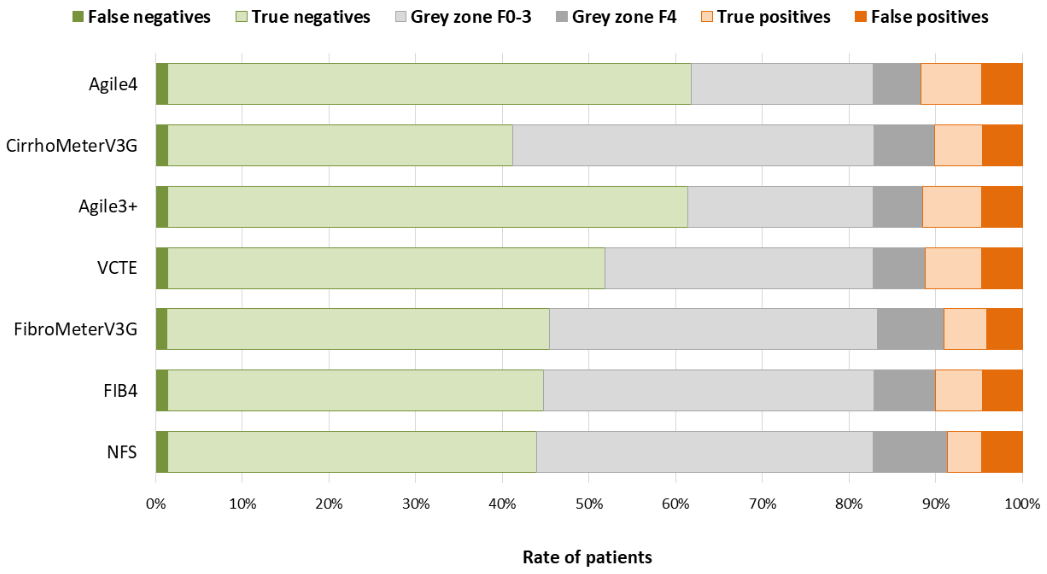

B: F34 Thresholds

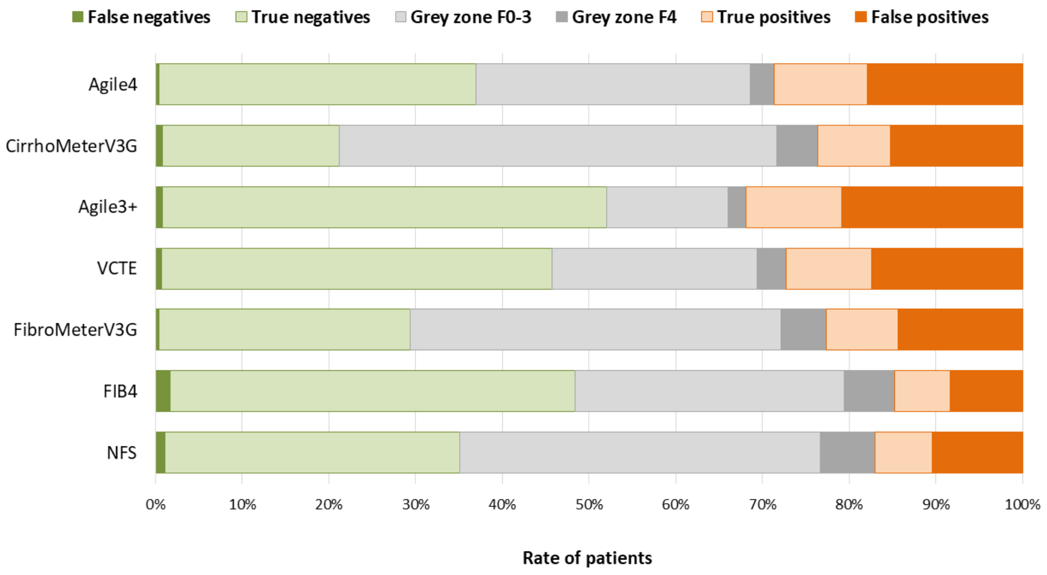

Supplementary Figure s4: Rates of false negatives, true negatives, true positives, and false positives for the diagnosis of cirrhosis using F4 thresholds (panel s4a) or F34 thresholds (panel s4b) in the validation set.

Bars in dark and light green correspond to the patients ruled out for cirrhosis. Bars in light and dark grey correspond to the patients in the grey zone between the rule out and the rule in thresholds. Bars in light and dark orange correspond to the patients rule in for cirrhosis.

NFS: NAFLD fibrosis score; VCTE: vibration controlled transient elastography.

A: F4 Thresholds

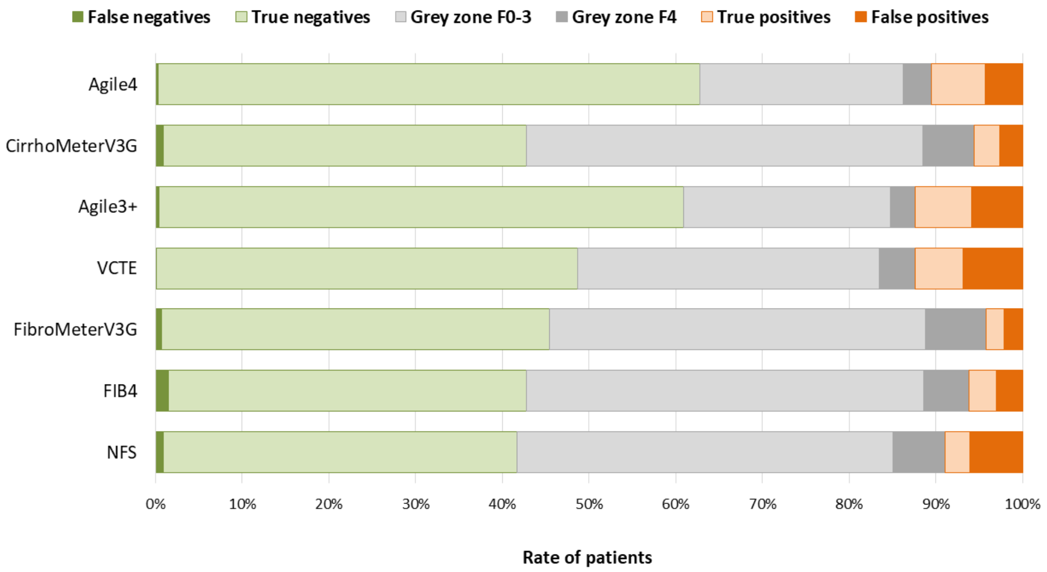

B: F34 Thresholds

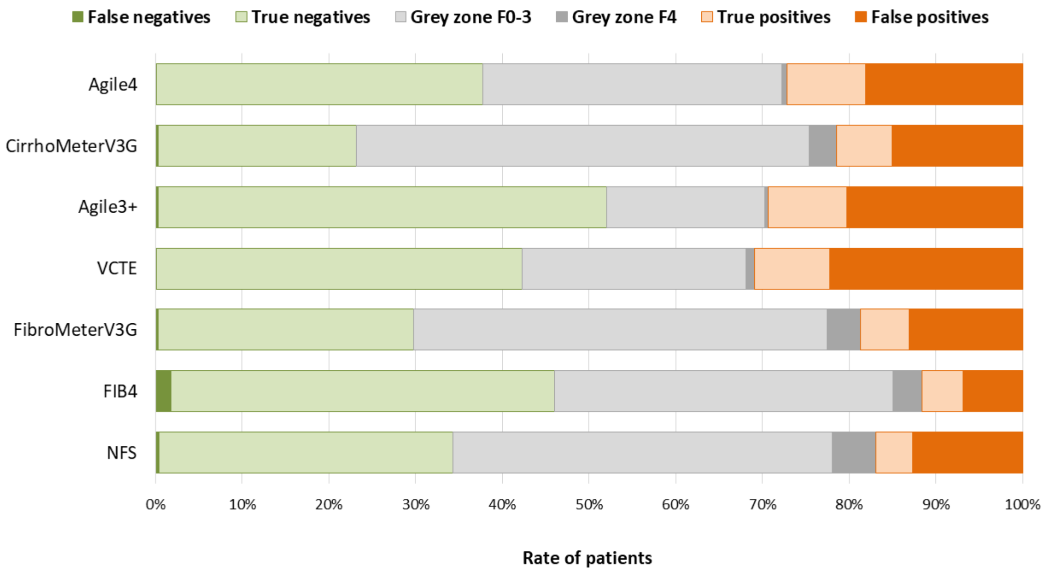

Supplementary Figure s5: Correlation between FibroMeter^{V3G} and CirrhoMeter^{V3G}, and between Agile 3+ and Agile4 in the derivation and validation sets.

DERIVATION SET

VALIDATION SET

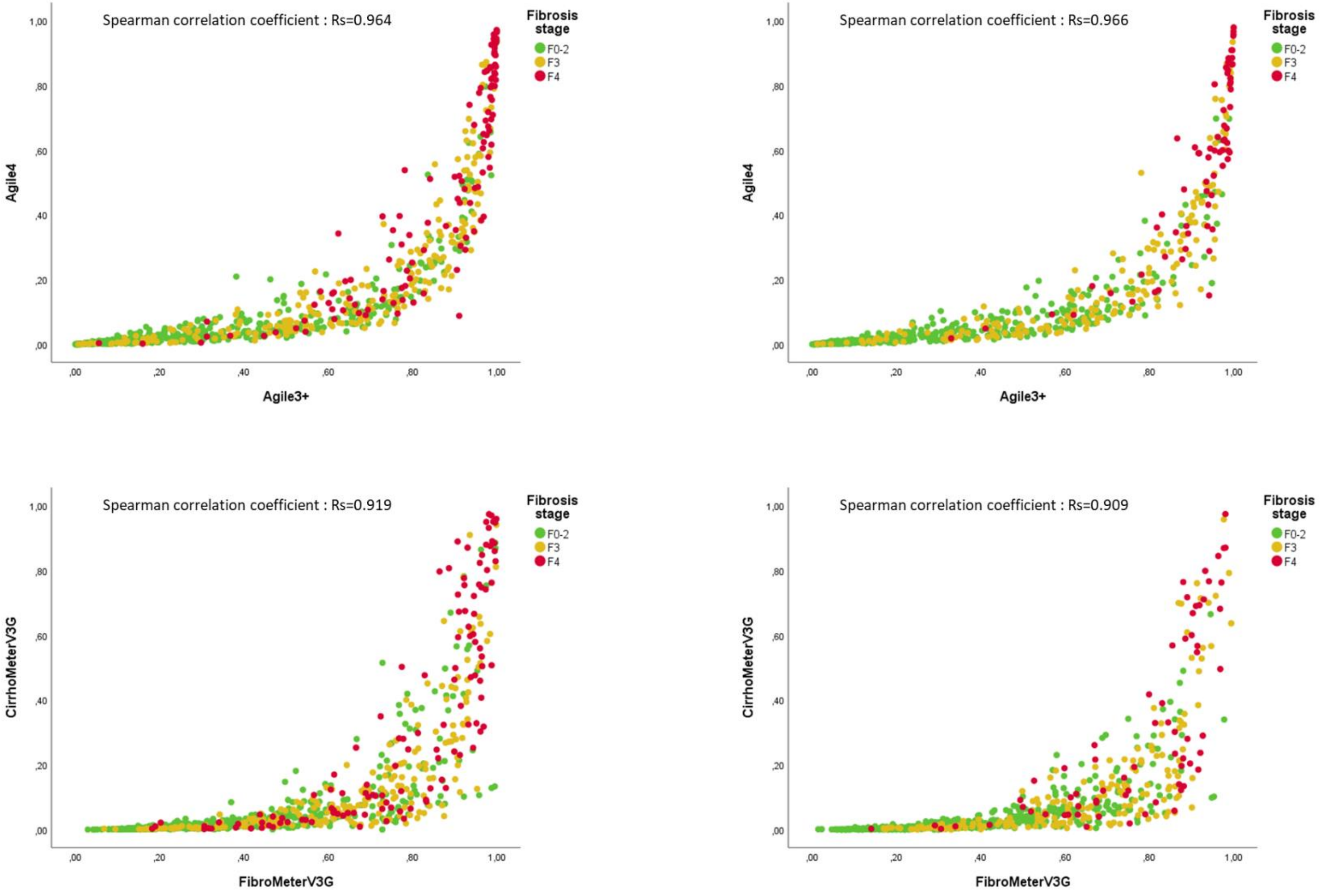

Supplementary Figure s6: Fibrosis stages as a function of 16 subgroups defined by the crossing of Agile3+/4 and FibroMeter^{V3G}/CirrhoMeter^{V3G} (FM/CM) classifications in the derivation set.

Source data are provided as a Source Data file.

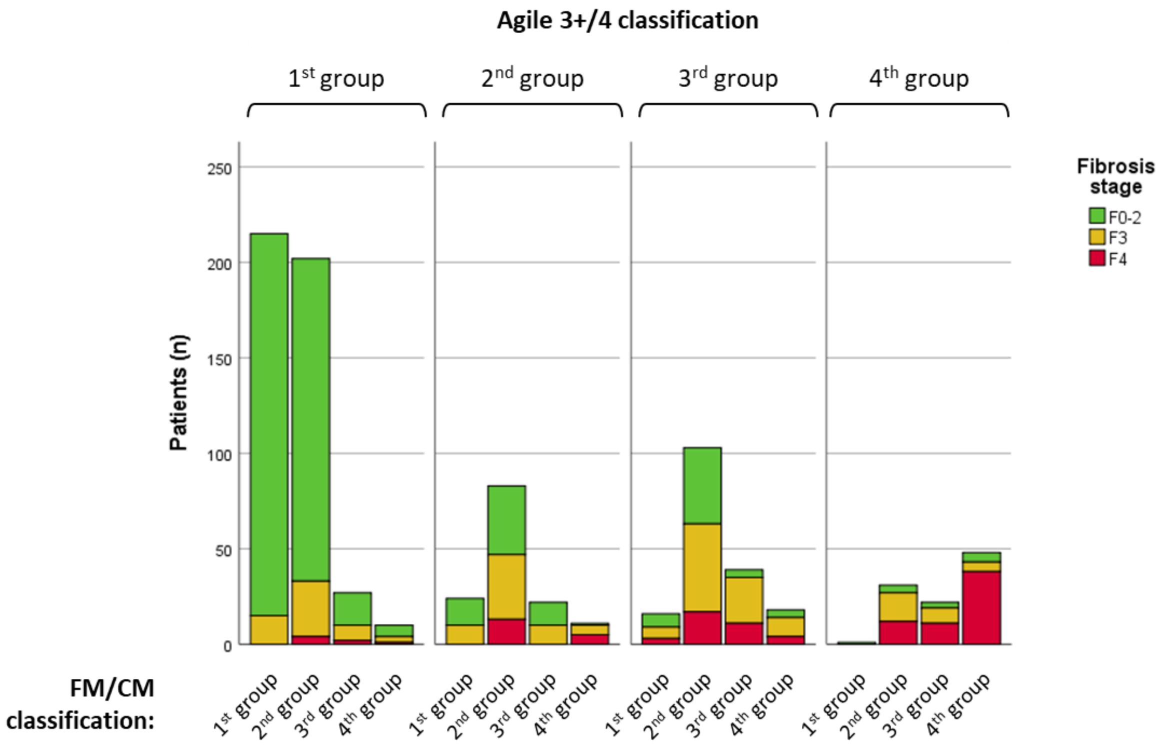

Supplementary Figure s7: Diagnostic pathway proposed by the latest EASL guidelines for the specialist area, based on agreement between non-invasive tests.

In the specialist area, the EASL guidelines recommend to first use vibration controlled transient elastography (VCTE). Then, a specialized blood test is recommended to confirm the diagnosis of liver fibrosis made with VCTE. The specialized blood test available for the study was the FibroMeter.

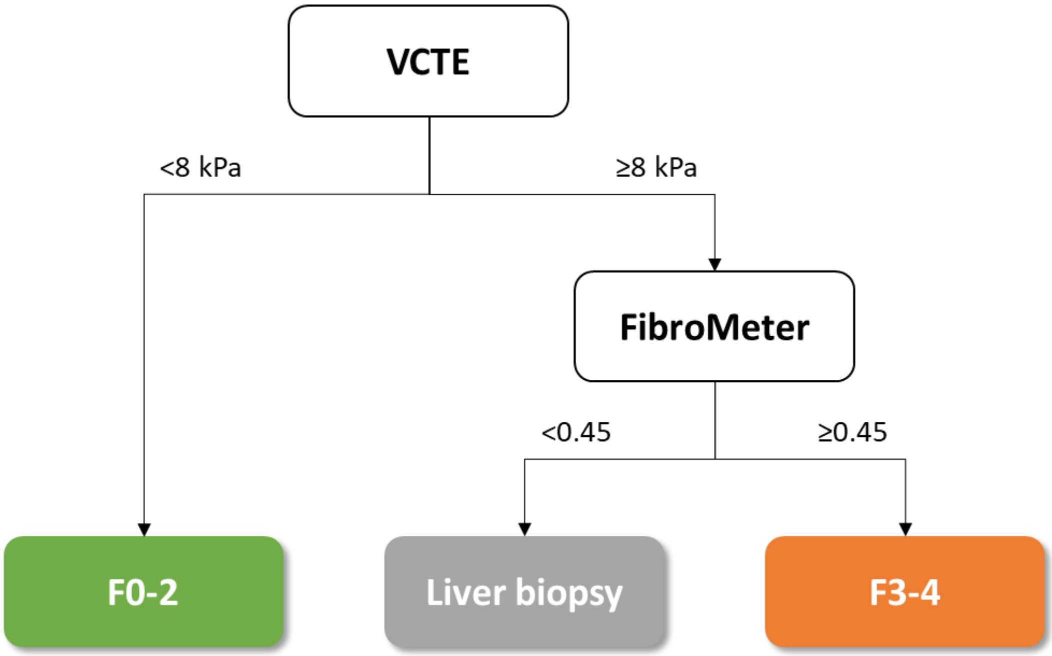

Supplementary Figure s8: Area of fibrosis measured by morphometry as a function of fibrosis stage. Morphometry was available in patients from Angers centre (validation set of the study). The centre line of the box corresponds to the median; the boundaries of the box correspond to the upper and lower quartiles; the limits of the whiskers correspond to the highest and lowest values included within a distance of 1.5 IQR.

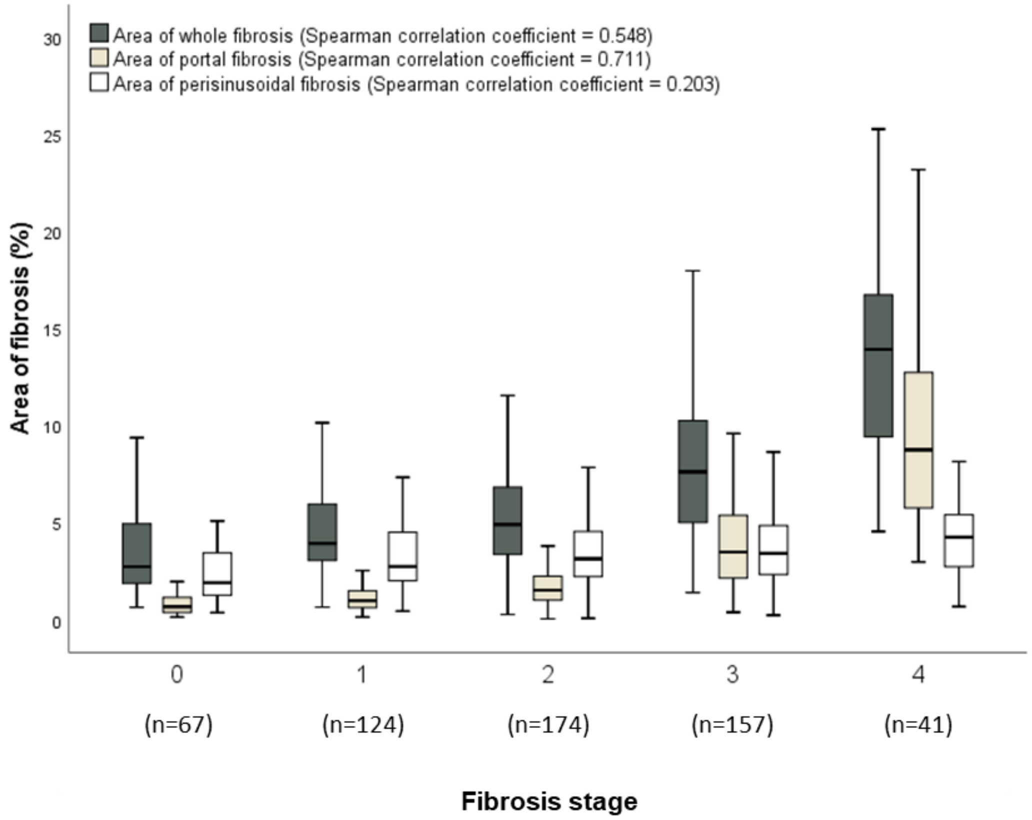

Fibrosis stage	Area of whole fibrosis (%)	Area of portal fibrosis (%)	Area of perisinusoidal fibrosis (%)
F0	3.7 (3.1-4.3)	0.9 (0.7-1.1)	2.7 (2.2-3.2)
F1	4.6 (4.2-5.0)	1.3 (1.1-1.4)	3.3 (3.0-3.7)
F2	5.3 (4.9-5.7)	1.8 (1.6-2.0)	3.5 (3.2-3.7)
F3	7.9 (7.3-8.4)	4.1 (3.7-4.5)	3.8 (3.5-4.1)
F4	14.2 (12.1-16.3)	10.1 (8.2-11.9)	4.1 (3.5-4.8)

Supplementary Figure s9: Area of fibrosis measured by morphometry on liver biopsies from Angers centre (validation set).

Panel s9a: Area of whole fibrosis as a function of the four groups defined by the study algorithm. The centre line of the box corresponds to the median; the boundaries of the box correspond to the upper and lower quartiles; the limits of the whiskers correspond to the highest and lowest values included within a distance of 1.5 IQR. Groups were compared using unidirectional ANOVA with Bonferroni correction for multiple comparisons. Panel s9b: Area of portal fibrosis as a function of the four groups defined by the study algorithm. Groups were compared using unidirectional ANOVA with Bonferroni correction for multiple comparison. Panel s9c: Area of fibrosis measured by morphometry as a function of the predicted risk by the cirrhosis risk chart. Area of whole fibrosis and area of portal fibrosis were measured in the 7 groups delineated by the cirrhosis risk chart presented in Figure 4a. Panel s9d: Area of fibrosis measured by morphometry as a function of the predicted risk by the advanced fibrosis risk chart. Area of whole fibrosis and area of portal fibrosis were measured in the 10 groups delineated by the advanced fibrosis risk chart presented in Figure 4b.

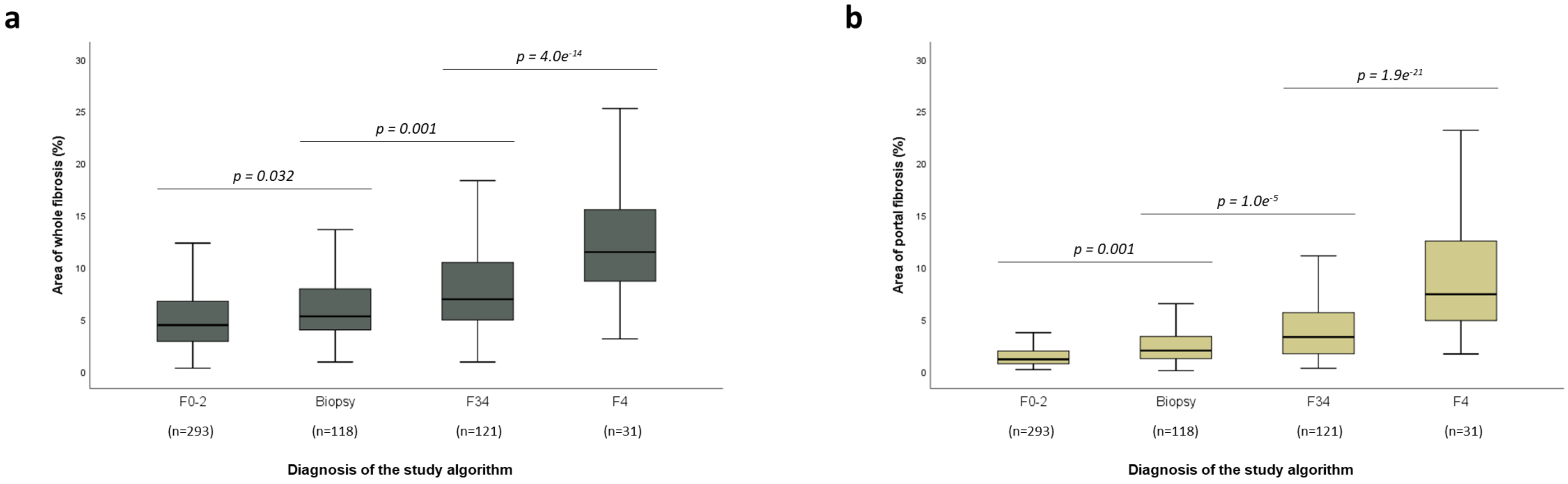

c

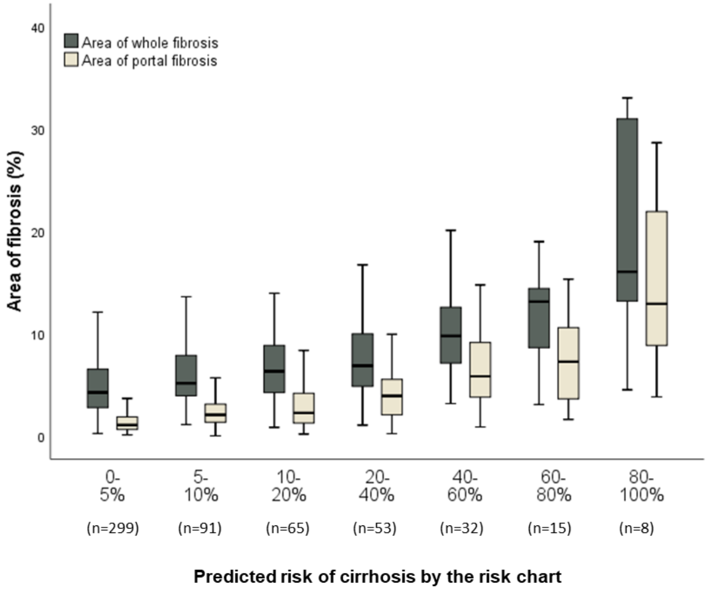

Area of whole fibrosis
Area of portal fibrosis
Area of fibrosis (%)
40
30
20
10
0
0-5%
5-10%
10-20%
20-40%
40-60%
60-80%
80-100%
(n=299)
(n=91)
(n=65)
(n=53)
(n=32)
(n=15)
(n=8)
Predicted risk of cirrhosis by the risk chart

d

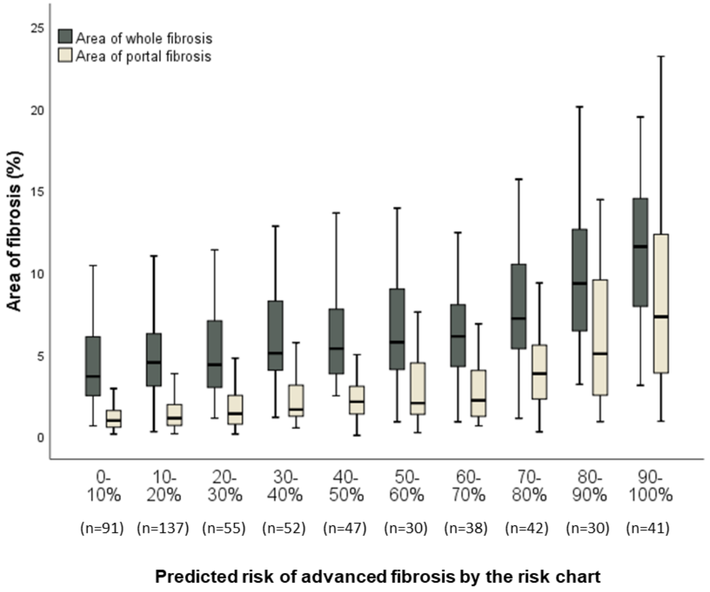

Area of whole fibrosis
Area of portal fibrosis
Area of fibrosis (%)
25
20
15
10
5
0
0-10%
10-20%
20-30%
30-40%
40-50%
50-60%
60-70%
70-80%
80-90%
90-100%
(n=91)
(n=137)
(n=55)
(n=52)
(n=47)
(n=30)
(n=38)
(n=42)
(n=30)
(n=41)
Predicted risk of advanced fibrosis by the risk chart

Supplementary Figure s10: Calibration of the predicted risk of cirrhosis by Agile4 and CirrhoMeter^{V3G} in the validation set.

Panel s10a: Calibration plot of the predicted risk of cirrhosis by Agile4. The blue dotted line represents perfect prediction (predicted risk of cirrhosis = observed prevalence of cirrhosis). The black solid line represents the observed prevalence of cirrhosis as a function of the predicted risk by Agile4.

Panel s10b: Calibration plot of the predicted risk of advanced fibrosis by CirrhoMeter^{V3G}.

a

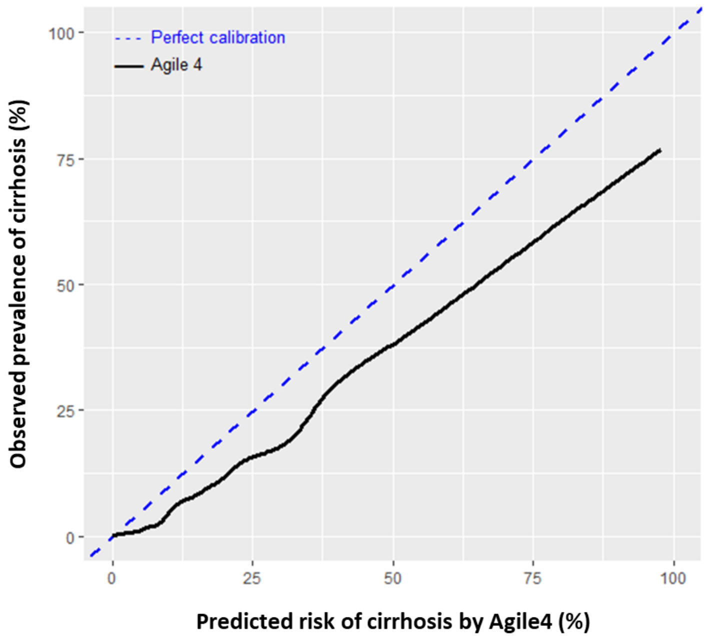

b

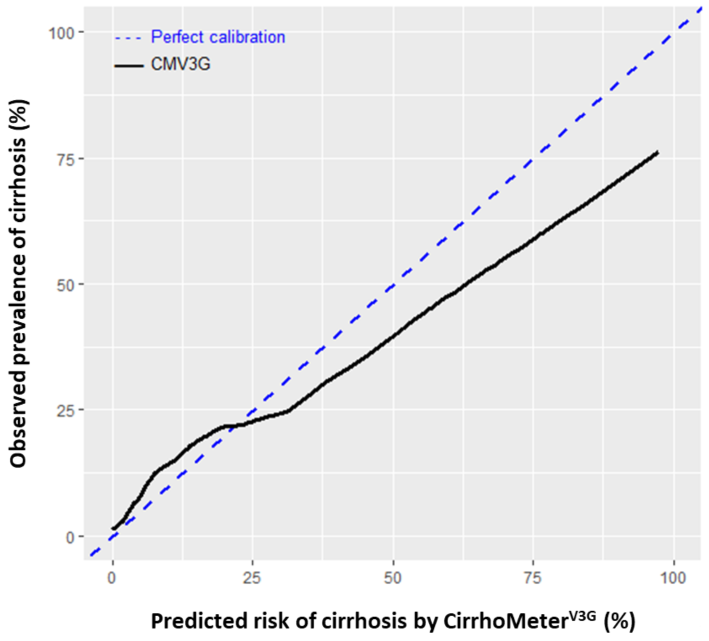

Supplement: Supplementary file 1 — Supplementary Information [file 41467_2023_40328_MOESM1_ESM.pdf]
