# Supplementary material for: Practical diagnosis of cirrhosis in non-alcoholic fatty liver disease using currently available non-invasive fibrosis tests
Source: Nat Commun. 2023 Aug 26;14:5219. doi: 10.1038/s41467-023-40328-4 (PMC10460420; doi:10.1038/s41467-023-40328-4)
Supplement: Supplementary file 3 — Reporting Summary [file 41467_2023_40328_MOESM3_ESM.pdf]

nature portfolio

Corresponding author(s): Jerome Boursier

Last updated by author(s): Jul 14, 2023

# Reporting Summary

Nature Portfolio wishes to improve the reproducibility of the work that we publish. This form provides structure for consistency and transparency in reporting. For further information on Nature Portfolio policies, see our Editorial Policies and the Editorial Policy Checklist.

## Statistics

For all statistical analyses, confirm that the following items are present in the figure legend, table legend, main text, or Methods section.

| n/a | Confirmed | |
|---|---|---|
| ☐ | ☒ | The exact sample size (*n*) for each experimental group/condition, given as a discrete number and unit of measurement |
| ☐ | ☒ | A statement on whether measurements were taken from distinct samples or whether the same sample was measured repeatedly |
| ☐ | ☒ | The statistical test(s) used AND whether they are one- or two-sided<br>*Only common tests should be described solely by name; describe more complex techniques in the Methods section.* |
| ☐ | ☒ | A description of all covariates tested |
| ☐ | ☒ | A description of any assumptions or corrections, such as tests of normality and adjustment for multiple comparisons |
| ☐ | ☒ | A full description of the statistical parameters including central tendency (e.g. means) or other basic estimates (e.g. regression coefficient) AND variation (e.g. standard deviation) or associated estimates of uncertainty (e.g. confidence intervals) |
| ☐ | ☒ | For null hypothesis testing, the test statistic (e.g. *F*, *t*, *r*) with confidence intervals, effect sizes, degrees of freedom and *P* value noted<br>*Give P values as exact values whenever suitable.* |
| ☒ | ☐ | For Bayesian analysis, information on the choice of priors and Markov chain Monte Carlo settings |
| ☒ | ☐ | For hierarchical and complex designs, identification of the appropriate level for tests and full reporting of outcomes |
| ☒ | ☐ | Estimates of effect sizes (e.g. Cohen's *d*, Pearson's *r*), indicating how they were calculated |

*Our web collection on statistics for biologists contains articles on many of the points above.*

## Software and code

Policy information about availability of computer code

| | |
|---|---|
| Data collection | This study utilised four cohorts of adult patients with NAFLD, liver biopsy, and VCTE examination. Three of them were local and independent cohorts from three French University Hospitals (Angers, n=579; Bordeaux, n=525; and Grenoble, n=117), as previously published (ref#27) and updated for the present work. The fourth cohort (n=347) came from a multicentre study performed in 7 liver centres across the United Kingdom (ref#28). Data from the four cohorts were pooled in an excel file (Excel v2303, Microsoft, Redmond, WA, USA). |
| Data analysis | Statistical analyses were performed using SPSS version 25.0 software (IBM, Armonk, NY, USA) and R version 3.6.2. |

For manuscripts utilizing custom algorithms or software that are central to the research but not yet described in published literature, software must be made available to editors and reviewers. We strongly encourage code deposition in a community repository (e.g. GitHub). See the Nature Portfolio guidelines for submitting code & software for further information.

## Data

Policy information about availability of data

All manuscripts must include a data availability statement. This statement should provide the following information, where applicable:

- Accession codes, unique identifiers, or web links for publicly available datasets
- A description of any restrictions on data availability
- For clinical datasets or third party data, please ensure that the statement adheres to our policy

Data that support the findings of this study come from four independent cohorts in different countries (France, UK) with different authorization processes for

delivery and sharing. The study data are available from the corresponding author (Pr Jerome Boursier, JeBoursier@chu-angers.fr) upon reasonable request, and after validation by all investigating centres.

## Research involving human participants, their data, or biological material

Policy information about studies with human participants or human data. See also policy information about sex, gender (identity/presentation), and sexual orientation and race, ethnicity and racism.

| | |
|---|---|
| Reporting on sex and gender | Sex and gender of participants were determined based on self-report. This work considered male/female sex only for the description of the patient characteristics (presented in Table 1). Neither sex nor gender was considered in the study design. |
| Reporting on race, ethnicity, or other socially relevant groupings | No data about race, ethnicity, or other socially relevant groupings has been provided by the investigating centres for the study. |
| Population characteristics | All patients underwent liver biopsy as part of investigation of NAFLD after exclusion of concomitant steatosis-inducing drugs (such as corticosteroids, tamoxifen, amiodarone, or methotrexate), excessive alcohol consumption (>30 g/day in men or >20 g/day in women), chronic hepatitis B or C infection, or evidence of other concomitant chronic liver disease. Patients were not included if they had presence or history of liver-related complication (ascites, variceal bleeding, jaundice, encephalopathy, hepatocellular carcinoma). Exclusion criteria for the present study were liver biopsy length <10 mm, VCTE failure, and missing biomarkers for blood fibrosis tests calculation. All patients came from hepatology clinics and none of the biopsies were performed during bariatric surgery.<br>Population characteristics are described in Table 1. |
| Recruitment | This study utilised four cohorts of adult patients with NAFLD, liver biopsy, and VCTE examination. Three of them were local and independent cohorts from three French University Hospitals (Angers, n=579; Bordeaux, n=525; and Grenoble, n=117), as previously published (ref#27) and updated for the present work. The fourth cohort (n=347) came from a multicentre study performed in 7 liver centres across the United Kingdom (ref#28). |
| Ethics oversight | All four cohorts obtained approval from Ethics Committees: CPP Ouest II Angers (CB2010-01) for Angers cohort; CPP Sud-Ouest et Outre Mer III for Bordeaux cohort; ARS Rhone Alpes (AC-2014-2094) for Grenoble cohort; and North Wales Research Ethics Committee (13/WA/0385) for UK cohort. All patients gave written informed consent before inclusion. |

Note that full information on the approval of the study protocol must also be provided in the manuscript.

# Field-specific reporting

Please select the one below that is the best fit for your research. If you are not sure, read the appropriate sections before making your selection.

☒ Life sciences ☐ Behavioural & social sciences ☐ Ecological, evolutionary & environmental sciences

For a reference copy of the document with all sections, see nature.com/documents/nr-reporting-summary-flat.pdf

# Life sciences study design

All studies must disclose on these points even when the disclosure is negative.

| | |
|---|---|
| Sample size | No sample size calculation was made. Indeed, the aim of the study was not to demonstrate a gain in accuracy for the diagnosis cirrhosis thanks to a fibrosis test, but rather to show how to use existing tests for the diagnosis of cirrhosis. Moreover, cohorts collected for the study provided large datasets for derivation (n=872) and validation (n=676).<br>We did, however, ensure that the study design was consistent with a phase III according to TRIPOD recommendations. The three French cohorts were not part of the development of the fibrosis tests evaluated in this study, whereas the UK cohort was part of the multicentre set in which Agile3+ and Agile4 fibrosis tests were developed. In addition, morphometry data (area of fibrosis) was only available for patients included from the Angers centre. We therefore decided to pool the UK and Bordeaux cohorts as a derivation set for our study, and the Angers and Grenoble cohorts as a validation set. This design allowed for: (i) a validation set that did not include any patient used for the derivation of the different fibrosis tests evaluated; (ii) a phase 3 design according to TRIPOD recommendations; (iii) well-balanced derivation (n=872) and validation (n=696) sets; and (iv) the use of morphometry as an additional evaluation measure in the validation set. |
| Data exclusions | Exclusion criteria for the present study were liver biopsy length <10 mm, VCTE failure, and missing biomarkers for blood fibrosis tests calculation (see the study flow chart in Supplementary Figure s1). |
| Replication | The study includes a derivation set and a validation set that were independent, making our work as a phase III study according to TRIPOD recommendations. The study algorithm and the risk prediction charts were developed in the derivation set. We then used the validation set to verify that the diagnostic accuracy of these tools was maintained in an independent set of patients. |
| Randomization | No randomization was required for the study, as it was a diagnostic study. It should be noted that fibrosis test results were not significantly different between derivation and validation sets.<br>Diagnostic studies do not control for potential confounding factors between derivation and validation sets (as most of the statistical tests used in these studies do not allow this). The most important in diagnostic studies is to verify that the accuracy observed in the derivation set is reproduced in the validation set comprising an independent set of patients. |
| Blinding | Pathological examinations were performed in each of the three French centres by a same senior expert specialized in hepatology (SM, BLB, |

| | |
|---|---|
| Blinding | NS) and blinded to patient data.<br>In the UK cohort, histological slides were analysed independently by two expert pathologists (PB, VP) who were blinded to each other's reading and to patient data.<br>Operator for liver stiffness measurements were blinded to histological and biological results. |

# Reporting for specific materials, systems and methods

We require information from authors about some types of materials, experimental systems and methods used in many studies. Here, indicate whether each material, system or method listed is relevant to your study. If you are not sure if a list item applies to your research, read the appropriate section before selecting a response.

## Materials & experimental systems

| n/a | Involved in the study |
|---|---|
| ☒ | ☐ Antibodies |
| ☒ | ☐ Eukaryotic cell lines |
| ☒ | ☐ Palaeontology and archaeology |
| ☒ | ☐ Animals and other organisms |
| ☐ | ☒ Clinical data |
| ☒ | ☐ Dual use research of concern |
| ☒ | ☐ Plants |

## Methods

| n/a | Involved in the study |
|---|---|
| ☒ | ☐ ChIP-seq |
| ☒ | ☐ Flow cytometry |
| ☒ | ☐ MRI-based neuroimaging |

## Clinical data

Policy information about clinical studies

All manuscripts should comply with the ICMJE guidelines for publication of clinical research and a completed CONSORT checklist must be included with all submissions.

| | |
|---|---|
| Clinical trial registration | Retrospective analysis of already published cohorts with their own registration (reference provided in the text manuscript). |
| Study protocol | Not available, as this was a retrospective analysis of already available and established patient cohorts. |
| Data collection | All cohorts have been already published, with references provided in the manuscript. |
| Outcomes | The primary outcome was cirrhosis as defined on histological reading. |
